# Exploring the Team Climate of Health and Social Care Professionals Implementing Integrated Care for Older People in Europe

**DOI:** 10.5334/ijic.5467

**Published:** 2020-10-19

**Authors:** Julie MacInnes, Erica Gadsby, Jillian Reynolds, Nuri Cayuelas Mateu, Manon Lette, Christina Ristl, Jenny Billings

**Affiliations:** 1 Centre for Health Services Studies, University of Kent, Canterbury, UK; 2 Agency for Health Quality and Assessment of Catalonia (AQuAS), Department of Health of the Catalan Government, Barcelona, ES; 3 Amsterdam University Medical Centres, VU University, Amsterdam Public Health research institute, Amsterdam, NL; 4 National Institute for Public Health and the Environment, Centre for Nutrition, Prevention and Health Services research, Bilthoven, NL; 5 Austrian Interdisciplinary Platform on Ageing/ÖPIA, AT

**Keywords:** team climate, integrated care, older people

## Abstract

**Background and problem statement:**

Team climate describes shared perceptions of organisational policies, practices and procedures. A positive team climate has been linked to better interprofessional collaboration and quality of care. Most studies examine team climate *within* health or social care organisations. This study uniquely explores the team climate of integrated health and social care teams implementing integrated care initiatives for older people in thirteen sites across seven European countries, and examines the factors which contribute to the development of team climate.

**Theory and methods:**

In a multiple case study design, data collected as part of the European SUSTAIN (Sustainable Tailored Integrated Care for Older People in Europe) project were analysed. The short-form Team Climate Inventory (TCI-14) was administered before and after implementation of the integrated care initiatives. Qualitative data was used to explain the changes in TCI-14 scores over time.

**Results and discussion:**

Overall, team climate was found to be high and increased over time in eight of the thirteen sites. The development of a shared vision was associated with a strong belief in the value and feasibility of the initiative, clear roles and responsibilities, and a reflective approach. Strong inter-personal relationships, shared decision-making, and high levels of commitment and motivation contributed to the development of participative safety. Support for innovation increased when staff had the ‘space’ and time to work together.

**Conclusion:**

This mixed methods study offers significant insights into the development and maintenance of team climate in complex, integrated care systems in Europe.

## Introduction

The dominant approach to understanding team climate focuses on shared perceptions of individuals. Organisational climate can be defined as ‘the shared perception of the way things are around here. More precisely, climate is shared perceptions of organisational policies, practices, and procedures’ [1]. Team climate is applied at the level of a proximal work group – that is, a permanent or semi-permanent group to which individuals are assigned, with whom they identify, and with whom they interact on a regular basis to perform work-related tasks. Several conditions are necessary for the development of shared perceptions, namely: that individuals interact at work; that there exists some common goal or attainable outcome which predisposes individuals towards collective action; and there is sufficient task interdependence such that individuals need to develop shared understanding and expected patterns of behaviour [1]. A positive team climate has been linked to better interprofessional collaboration as well as quality of care outcomes including access to services, continuity of care, clinical management of long term conditions and patient satisfaction [2].

Team climate has been commonly measured by the Team Climate Inventory (TCI), developed by Anderson and West [1]. The TCI has been validated and applied in a variety of settings including primary and secondary care and in a number of countries. It consists of four domains or sub-scales: ‘Vision’ represents the perceived clarity, agreement with and attainability of the team’s objectives; ‘Participative Safety’ describes team members’ psychological safety, sharing of information and participation in decision-making; ‘Task Orientation’ refers to members’ commitment to high standards of performance and appraisal of weaknesses; and ‘Support for Innovation’ measures the perceived co-operation and help in applying new ideas and improvement. The TCI has been further developed by Kivimaki and Elovainio [3] into a short version consisting of 14 items across the four domains – the TCI-14.

A number of studies have used the TCI to determine relationships between team climate and individual and team characteristics, and interprofessional collaboration in primary care settings [4,5,6,7]. However, most studies have used the TCI to consider team climate *within* organisations, particularly amongst healthcare staff in primary care settings. Few have examined team climate *across* organisations, especially those where proximal work groups exist across organisations as occurs in integrated care. As a result, little is known about the team climate of interprofessional teams across culturally distinct health and social care organisations.

This paper draws on data collected as part of the European SUSTAIN (Sustainable Tailored Integrated Care for Older People in Europe) project. SUSTAIN was a four-year research project (2015–2019) which aimed to support and monitor improvements to integrated care services for older people living at home with multiple health and social care needs, and in so doing move towards more person-centred, prevention-oriented, safe and efficient care [8].

This aim of this paper is to explore the team climate of health and social care teams implementing integrated care initiatives for older people in Europe, and examines the factors which contribute to the development of team climate.

Specific objectives are to:

- Measure the team climate before and after the implementation of integrated care initiatives at the thirteen SUSTAIN sites within seven European countries, using the TCI-14
- Determine if there are any differences in TCI-14 scores before and after implementation at the sites
- Explore the reasons for any changes in TCI-14 scores using data from qualitative interviews and focus groups with health and social care professionals, and contextual data drawn from in-depth case studies.

## Theory and methods

### Overall SUSTAIN methodology

The SUSTAIN project evaluated and compared improvements to integrated care services implemented in thirteen participating sites in Austria, Estonia, Germany, The Netherlands, Norway, Spain (Catalonia) and the United Kingdom. The target population were older people living at home with complex health and social care needs. Although the target group was the same, the initiatives provided a varied range of services in different care settings (Table 1).

**Table 1:** Characteristics of the thirteen integrated care sites participating in the SUSTAIN project.

Country	Site-specific Identifier	Location	Integrated care initiative	Type of care service

**Austria**	AT1	Vienna	Gerontopsychiatric Centre	Dementia care
**Estonia**	EST1	Idu-Viru	Alutaguse Care Centre	Home nursing and rehabilitative care
	EST2	Tallinn	Medendi	Home nursing
**Germany**	GER1	Uckermark	KV RegioMed Zentrum Templin	Rehabilitative care
	GER2	Berlin Marzahn-Hellersdorf	Careworks Berlin	Home nursing and rehabilitative care
**The Netherlands**	NL1	West-Friesland	Geriatric Care Model	Proactive primary care
	NL2	Arnhem	Good in one Go	Transitional care
**Norway**	NO1	Surnadal	Holistic Patient Care at Home	Home nursing and rehabilitative care
	NO2	Sondre Nordstrand, Oslo	Everyday Mastery Team	Rehabilitative care and mastery of activities of daily living
**Spain (Catalonia)**	SP1	Osona	Severe Chronic Patients/Advanced chronic disease/Geriatrics	Proactive primary and intermediate Care
	SP2	Sabadell	Social and health care integration	Proactive primary care
**United Kingdom**	UK1	Kent	Over 75 Service	Proactive primary care
	UK2	Kent	Swale Home First	Transitional care

Guided by the Evidence Integration Triangle [9], the SUSTAIN project adopted an implementation science approach in order to improve current practice. Local stakeholders and researchers worked closely together to co-design and implement a wide range of activities, aiming to improve the person-centeredness, prevention-orientation, safety, co-ordination and efficiency of integrated working. Using a multiple embedded case study design [10], each site was considered one case study. Qualitative and quantitative data from different sources were collected, analysed and reported at each site. Case study reports consisted of an analysis of case study data and overall conclusions and recommendations at a country-wide level [11]. Finally, all findings were combined into an overarching analysis to provide insights into what worked and what did not work in improving integrated care for older people in Europe. A more detailed description of the SUSTAIN project’s approach can be found in a previous paper [8].

### Data collection

For this paper, the following SUSTAIN data sources were used:

1. *TCI-14 and demographic questionnaire*: At each case site, participating health and social care professionals and managers completed the TCI-14 and a demographic questionnaire which provided information on gender, age, educational level, professional discipline and contractual status. The TCI-14 was completed at the start and end of the implementation period to capture changes in team climate over an 18 month period.
2. *Site-specific templates explaining the TCI-14 data*: Each research team who had worked with an individual case site, was asked to provide qualitative explanations and illustrative quotes to explain the TCI-14 findings at each site. The researchers had worked closely with a site in their own country, and so were best placed to provide additional insights and explanations for any changes in team climate. Researchers drew their knowledge and experience from interviews and focus groups with professionals and managers, minutes of meetings and field notes collected at each site.
3. *Site case study reports*: Additional data, illustrating explanatory factors for any changes in team climate over time were extracted from the individual case study reports.

### Data analysis

Demographic data for the full study population were summarised in terms of absolute and relative frequencies.

Outcomes data were analysed by quantitative and qualitative methods.

#### Quantitative analysis of TCI-14 scores

For each site the change in TCI-14 scores was summarized by calculating the mean scores at baseline and at follow-up, as well as their difference. The null hypothesis of no mean difference was tested using a linear mixed model with time-point as fixed effect and subject as random effect. This model is suitable to utilize the combined data from subjects who were observed at both time-points and from subjects who were observed at one time-point only (e.g. new team members). To account for the relatively small sample size the tests were calculated using the degrees of freedom method [12]. An analogous analysis was applied to compare the TCI-14 sub-scales of Vision, Participative Safety, Task Orientation and Support for Innovation between baseline and follow up. P-values <0.05 were considered significant differences.

#### Qualitative analysis of the site specific templates and case study reports

Qualitative analysis of the site-specific templates explaining the TCI-14 data and extracts from individual case study reports was guided by the framework analysis method [13]. A thematic framework was developed based on themes identified in the literature and those inductively derived from the data. For each theme, factors enhancing team climate and factors inhibiting team climate were identified. The thematic framework was used to assign codes to relevant passages of the qualitative data. Two researchers coded the data and cross-checked each other’s work to identify any discrepancies in criteria, solve doubts and agree on the final coding of all data extracts. Identified patterns and (sub)themes were then developed into thematic statements.

Results from the qualitative analyses were synthesized and compared in order to study patterns and inconsistencies, and find explanations for changes in the TCI-14 data during the implementation of the integrated care initiatives.

### Ethical statement

Ethical approval was granted by the ethical review committees of Estonia, Norway, Spain (Catalonia) and the United Kingdom. In Austria, Germany, and the Netherlands research activities were exempt from the need for a formal ethics committee review according to national standards and regulations. Prior to data collection, informed consent was obtained for all study participants. Confidentiality and anonymity was assured for all participants by assigning individual identity codes.

## Results

### Demographic characteristics of the managers and professionals

In total, 244 staff members completed the demographic questionnaire. Demographic characteristics are described in Table 2.

**Table 2:** Demographic characteristics of managers and professionals.

	Number(N=)	Percentage of Total(%)

**Country (N = 244)**		
Austria	12	4.9
Estonia	26	10.7
Germany	23	9.4
Netherlands	22	9.0
Norway	33	13.5
Spain (Catalonia)	75	30.7
United Kingdom	53	21.7
**Gender (N = 242)**		
Female	208	86
Male	34	14
**Age (years) (N = 239)**		
18–24 years	2	0.8
25– 34 years	42	17.6
35–44 years	73	30.5
45–54 years	66	27.6
55–64 years	55	23.0
65+	1	0.4
**Highest Educational level (N = 236)**		
Secondary	19	8.0
Vocational	28	11.9
Graduate certificate	30	12.7
Graduate diploma	51	21.6
Bachelors degree	53	22.5
Master degree	42	17.8
Doctoral degree	8	3.4
Other	5	2.0
**Staff group (N = 244)**		
Managers (all)	36	14.8
Administrative & clerical	23	9.4
Allied health professional	32	13.1
Medical	35	14.3
Nursing	66	27.0
Social Work	44	18.0
Other (including voluntary)	8	3.3
**Working hours (N = 238)**		
Full-time	179	75.2
Part-time	59	24.8

#### Summary of TCI-14 scores

Table 3 shows the number of participants completing the TCI-14 at each site at baseline and follow-up.

**Table 3:** Number of staff completing the TCI-14.

Country	Site	Baseline (N=)	Follow-up (N=)

Austria	AT1	7	8
Estonia	EST1	7	15
	EST2	12	6
Germany	GER1	6	7
	GER2	10	10
Netherlands	NL1	10	10
	NL2	8	5
Norway	NO1	16	13
	NO2	9	13
Spain (Catalonia)	SP1	51	34
	SP2	11	10
United Kingdom	UK1	17	25
	UK2	7	4
Total		171	160

Mean total TCI-14 scores and mean sub-scores at baseline and follow-up are summarised in Table 4.

Overall, the mean total scores were positively skewed ranging from 3.25 (NL2) to 4.76 (EST1), out of a maximum 5.0 (Table 4).

**Table 4:** Total TCI-14 Scores and sub-scale scores at each site.

Country	Site	Total TCI/Sub-scales	Mean (range) Baseline	Mean (range) Follow-up	Mean Difference	Effect size	Significance

**Austria**	**AT1**	Total TCI	3.67 (3.00–4.36)	4.21 (2.75–4.93)	0.54	0.33	0.13
		Vision	3.75 (2.75–4.50)	4.09 (3.00–4.75)	0.34	0.37	0.37
		Participative Safety	3.79 (3.00–4.75	4.31 (2.50–5.00)	0.53	0.4	0.21
		Task Orientation	3.81 (3.33–5.00)	4.25 (3.00–5.00)	0.44	0.39	0.27
		Support for Innovation	3.29 (3.00–4.00)	4.21 (2.67–5.00)	0.92	0.33	*0.01
**Estonia**	**EST1**	Total TCI	4.76 (4.57–4.86)	4.07 (3.36–4.86)	–0.68	0.12	*<0.01
		Vision	4.75 (4.75–4.75)	4.20 (3.25–5.00)	–0.55	0.13	*<0.01
		Participative Safety	4.68 (4.50–5.00)	4.20 (3.25–5).00	–0.48	0.16	*<0.01
		Task Orientation	4.76 (4.33–5.00)	3.87 (3.00–5.00)	–0.90	0.19	*<0.01
		Support for Innovation	4.86 (4.67–5.00)	3.93 (3.00– 5.00)	–0.92	0.20	*<0.01
	**EST2**	Total TCI	4.52 (3.64–5.00)	4.28 (3.71–4.79)	–0.24	0.19	0.23
		Vision	4.65 (4.25–5.00)	4.38 (4.00– 4.75)	–0.27	0.16	0.13
		Participative Safety	4.54 (4.00–5.00)	4.35 (3.75–4.75)	–0.19	0.18	0.30
		Task Orientation	4.19 (2.67–5.00)	3.89 (3.00–4.67)	–0.31	0.40	0.45
		Support for Innovation	4.64 (3.33–5.00)	4.44 (4.00–5.00)	–0.19	0.22	0.39
**Germany**	**GER1**	Total TCI	4.64 (4.43–4.93)	4.33 (4.00–4.71)	–0.32	0.11	*0.01
		Vision	4.62 (4.25–4.75)	4.46 (4.25–4.75)	–0.16	0.11	0.16
		Participative Safety	4.96 (4.75–5.00)	4.50 (4.00–5.00)	–0.46	0.14	*0.01
		Task Orientation	4.44 (3.67–5.00)	3.86 (3.33–4.67)	–0.59	0.30	0.07
		Support for Innovation	4.44 (4.00–5.00)	4.38 (4.00–5.00)	–0.06	0.24	0.79
	**GER2**	Total TCI	4.19 (3.64–4.64)	4.16 (3.86–4.57)	–0.03	0.14	0.81
		Vision	4.12 (3.75–4.50)	4.05 (3.75–4.75)	–0.08	0.13	0.58
		Participative Safety	4.40 (3.75–5.00)	4.45 (4.00– 4.75)	0.05	0.19	0.79
		Task Orientation	4.23 (3.67–5.00)	4.13 (3.33–5.00)	–0.10	0.21	0.64
		Support for Innovation	3.97 (3.00–4.67)	3.97 (3.00–4.67)	0.00	0.24	1.00
**Netherlands**	**NL1**	Total TCI	3.25 (1.79–4.86)	3.56 (2.07–4.21)	0.31	0.33	0.36
		Vision	3.62 (1.50–4.75)	3.67 (2.25–4.50)	0.05	0.37	0.89
		Participative Safety	3.12 (1.75–5.00)	3.67 (1.50–4.25)	0.54	0.43	0.23
		Task Orientation	2.90 (1.00–5.00)	3.30 (2.67–4.00)	0.40	0.38	0.31
		Support for Innovation	3.27 (2.00–4.67)	3.37 (2.00–4.33)	0.10	0.32	0.75
	**NL2**	Total TCI	3.39 (3.00–3.80)	3.47 (3.07–3.93)	0.08	0.17	0.65
		Vision	3.00 (3.00– 3.00)	3.17 (3.00–3.50)	0.17	0.13	0.42
		Participative Safety	3.84 (3.00–4.50)	4.00 (3.25–5.00)	0.16	0.35	0.66
		Task Orientation	2.83 (2.00–3.67)	3.07 (2.67–3.33)	0.23	0.22	0.30
		Support for Innovation	3.46 (3.00–4.00)	3.47 (3.00–4.00)	0.01	0.24	0.97
**Norway**	**NO1**	Total TCI	3.85 (2.93–4.64)	3.95 (3.14–4.64)	0.10	0.19	0.61
		Vision	4.11 (3.00–4.75)	4.23 (3.75–4.75)	0.12	0.16	0.45
		Participative Safety	3.83 (2.50–4.75)	3.90 (3.25–5.00)	0.07	0.23	0.75
		Task Orientation	3.58 (2.33–4.67)	3.67 (2.33–4.67)	0.08	0.27	0.76
		Support for Innovation	3.77 (3.00–4.67)	3.90 (2.67–5.00)	0.13	0.24	0.59
	**NO2**	Total TCI	3.77 (3.14–4.29)	3.63 (2.93–4.64)	–0.14	0.18	0.45
		Vision	3.92 (3.50–4.25)	3.62 (3.00–4.75)	–0.30	0.17	0.09
		Participative Safety	4.14 (3.5 0– 4.75)	4.00 (3.00–5.00)	–0.14	0.19	0.48
		Task Orientation	3.70 (2.67–5.00)	3.64 (3.00–4.67)	–0.06	0.27	0.82
		Support for Innovation	3.15 (2.33–4.33)	3.15 (1.67–4.33)	0.01	0.34	0.98
**Spain (Catalonia)**	**SP1**	Total TCI	3.82 (2.00–5.00)	4.04 (2.57– 5.00)	0.22	0.12	0.06
		Vision	3.99 (2.00–5.00)	4.29(2.25–5.00)	0.27	0.12	*0.02
		Participative Safety	4.03 (2.00–5.00)	4.15(2.25– 5.00)	0.12	0.14	0.39
		Task Orientation	3.74 (2.00–5.00)	3.96 (2.00–5.00)	0.23	0.16	0.16
		Support for Innovation	3.39 (1.67–5.00)	3.64 (2.00– 5.00)	0.26	0.16	0.11
	**SP2**	Total TCI	3.97 (3.54–4.71)	4.47 (4.00–4.75)	0.50	0.13	*0.01
		Vision	4.09 (3.50–5.00)	4.53 (3.75–5.00)	0.43	0.18	*0.02
		Participative Safety	4.18 (3.00–5.00)	4.72 (4.00–5.00)	0.54	0.2	*0.01
		Task Orientation	3.85 (3.00–4.33)	4.57 (4.00–5.00)	0.72	0.18	*0.01
		Support for Innovation	3.64 (2.33–5.00)	4.00 (3.67–4.67)	0.36	0.26	0.18
**United Kingdom**	**UK1**	Total TCI	3.97 (3.07–5.00)	4.10 (2.64–4.85)	0.14	0.2	0.49
		Vision	3.97 (3.00–5.00)	4.18 (2.75–4.75)	0.21	0.18	0.25
		Participative Safety	4.09 (3.00–5.00)	4.24 (2.75–5.00)	0.15	0.2	0.46
		Task Orientation	3.94 (2.67–5.00)	3.98 (2.33–5.00)	0.04	0.25	0.87
		Support for Innovation	3.82 (2.67–5.00)	3.93 (2.00–5.00)	0.11	0.26	0.68
	**UK2**	Total TCI	4.11 (3.64–4.64)	3.77 (3.43–4.14)	–0.34	0.23	0.17
		Vision	4.25 (4.00–4.50)	3.50 (3.00–3.75)	–0.75	0.2	*0.01
		Participative Safety	4.43 (3.75–5.00)	3.88 (3.75–4.00)	–0.55	0.19	*0.02
		Task Orientation	3.76 (3.00–4.67)	3.83 (3.00–5.00)	0.07	0.51	0.89
		Support for Innovation	3.86 (3.00–4.33)	3.92 (3.00–5.00)	0.06	0.49	0.90

* significant at p < 0.05.

Eight sites showed an increase in the mean total TCI-14 score from baseline to follow-up; of these one showed a statistically significant increase (SP2). In contrast five sites showed a decrease in the mean total TCI-14 score of which two showed a statistically significant decrease (EST1, GER2).

Sub-scale scores were similarly positively skewed with a lowest score of 2.83 (NL2 – Baseline, Task Orientation) and a highest score of 4.96 (GER1 – Baseline, Participative Safety). For Vision, seven sites showed an increase between baseline and follow-up of which two were statistically significant (SP1, SP2), and six showed a decrease, two of which was statistically significant (EST1, UK2). For Participative Safety, eight sites showed an increase of which one was statistically significant (SP2), and five showed a decrease, three of which were statistically significant (EST1, GER1, UK2). For Task Orientation, eight sites showed an increase of which one was statistically significant (SP2), and five showed a decrease of which one was statistically significant (EST1). Finally, for Support for Innovation, eight sites showed an increase of which only one was statistically significant (AT1), three sites showed a decrease of which one was statistically significant (EST1) with two sites showing no change (GER1, NOR2).

In summary, TCI-14 scores present a mixed picture with an overall tendency to increase between baseline and follow-up.

#### Explanation of TCI-14 findings using the qualitative data

This section examines the qualitative data to draw out possible explanations and contradictions for where TCI-14 scores increased, decreased or stayed the same. Themes identified from the literature and inductively from the data were: shared goals, norms and values; understanding roles and responsibilities; communication and decision-making processes; interpersonal relationships; individual or professional character traits; information sharing; clarity of planning; and organisational support, policies and procedures. It was found that themes did not map neatly onto the four TCI sub-scales, with most cutting across several sub-scales. Vision was most closely mapped to issues related to shared goals, norms and values, communication and decision making processes, clarity of planning, and organisational support, policies and procedures. Participative safety was most closely mapped to issues related to interpersonal relationships and communication and decision-making processes. The Task Orientation and Support for Innovation’ sub-scales were particularly difficult to associate with any one theme.

## Vision

For this sub-scale, a higher score represents agreement with objectives; a clear understanding of the team’s objectives; a sense that the objectives are achievable; and a feeling that the objectives are of worth to the organisation. Table 5 presents the thematic statements explaining the increase or decrease in Vision scores.

**Table 5:** Explanations for changes in the Vision sub-scale.

	Where Vision score increased (7 sites)	Where Vision score decreased (6 sites)

**Thematic statements**	Competent management/leadership, and key people with clear roles helped to push the initiative forward Strong collaboration to co-design the initiative and good continuity of team members over time Feedback to reinforce a shared vision and build confidence in the achievability of outcomes Careful planning to ensure feasibility, regular meetings, and additional measures to ensure good information sharing Maintenance of focus by declining participation in other projects Sense of urgency, political support, and support of management that enhanced motivation/enthusiasm	Weak or unstable leadership External constraints affecting the initiative Absence of clearly delineated roles and responsibilities for team members Lack of a strong communication plan, and failure to develop a common understanding of the initiative Focus of the initiative lost over time, or had to change to fit external constraints (leading to frustration).

The qualitative data revealed a complex picture in terms of Vision. Whilst there was a general sense of confidence in some integrated care initiatives, some staff remained sceptical about the potential impact and/or sustainability. Similarly, whilst staff might share a common vision, they might not share a clear idea of how that will be achieved. Interestingly, a lack of resources, and the struggle this might precipitate, did not necessarily impact on the team’s overall vision. Sometimes, initiatives evolved more slowly and it took time for a clear vision to be developed and commonly understood.

The qualitative data from sites where the Vision score increased showed that there was a strong belief that the initiative was valuable and important.

“No one was freed up time to do this; everyone has arranged a way to do it and everyone, in the time they had, has decided it was important and has done it” (SP1/M1)

“Knowing that what we’re doing is having an impact and that patients are benefiting from it, in that you’re not just doing something for the sake of doing something, there is sort of a purpose” (UK1/P6)

Key factors that contributed to a shared vision were associated with effective management and/or leadership, a collaborative approach, good planning, clear roles and responsibilities and a reflective approach. Conversely, data from sites where the Vision score decreased suggested that there was a lack of clarity around how the initiative was going to achieve its objectives, or a feeling that the objectives were not achievable. For example:

“There’s been so many changes to what the focus has been” (UK2/M5)

“We struggled quite a bit in the beginning, both in figuring out what we were participating in, and in defining the different roles […]. We spent a lot of time on this.” (NO2/M1)

Continuity of the team, especially those in a leadership role was also important, as illustrated in this quote for a site where the Vision score decreased:

“For me, [the initiative] has, because of the staffing situation, had its ups and downs. With its changing of project leaders, it has been difficult to maintain the continuity of it all. That has been something that has influenced the whole [initiative]. That we, from the beginning have not had continuity in leadership and continuity in the knowledge bearers” (NO2/M2)

## Participative Safety

A higher score for the Participative Safety sub-scale is associated with a ‘we are in it together’ attitude, and a team in which people keep each other informed, feel understood and accepted, and make real attempts to share information. Analysis of the qualitative data revealed some key explanations for an increase or a decrease in score, as shown in Table 6.

**Table 6:** Explanations for changes in the Participative Safety sub-scale.

	Where Participative Safety score increased (8 sites)	Where Participative Safety score decreased (5 sites)

**Thematic statements**	Space and time was given to meet regularly and focus on the initiative The contribution of different team/staff members was recognised, with a sense that they can improve services by combining perspectives and efforts (developing co-responsibility) Leadership was non-hierarchical or shared between team members, with decisions made collaboratively or by consensus Team members had previous experience of working together, and got to know each other better as the initiative went on Professionals and managers referred to each other in positive terms Team members were committed, listened to each other and were willing to help each other when difficulties arose A shared sense of urgency and presence of the user perspective acted as a binding factor for developing trust and understanding Managers were motivated, well connected to stakeholders, and contributed meaningfully and collaboratively alongside staff	Some partners (such as operational level staff, external stakeholders or the public) were not as involved as they could have been, or were involved rather late The number of different teams involved, and the fragmented nature of them (in different locations, with different geographical areas, and working on different systems and to different schedules) hindered good communication and integration A historical and cultural separation of ‘health’ and ‘social care’ made it difficult to overcome a sense of ‘us’ and ‘them’, with some stereotyping and resentment on both sides Limited capacity/resources from one partner more than the other, stifled their ability to co-lead the initiative Inconsistent and limited involvement of staff, unfilled posts and staff changes disrupted interpersonal relations Lack of a dedicated manager for a period of time Staff felt they had limited ability/capacity to contribute to elements of the initiative

The act of co-designing and implementing the integrated care initiative brought professionals together, strengthened relationships, helped them to understand each other’s roles and responsibilities, enabled initial tensions between different professional groups to be overcome, enhanced trust, and increased a sense of team identity.

Explanations for increases in this sub-scale focused on interpersonal relations, shared responsibility and decision-making, and high levels of commitment, motivation and involvement of different agencies including patient representatives.

“It’s that you feel comfortable to ask someone, because you know it’s something that is probably part of their job. Otherwise, you wonder sometimes about whom to go to. Now you know a little bit of what everyone is doing. And you know each other, so it’s not so bad if you ask the wrong question to the wrong person sometimes. Because then the other one will just tell you, no, you should go to him or her with that question. That is that feeling of safety and trust that you have” (NL1/P6)

“Establishing more flat roles, not the roles of one being the boss and others do…I think this has been one of the assets […] it has facilitated making everybody responsible” (SP1/M1)

In sites where the sub-scale decreased, there seemed to be partial or unequal involvement or contribution from some partners, as well as structural and/or historical and cultural factors that inhibited working together. Where involvement in the initiative was on top of an already busy workload, engagement and good communications were sometimes inhibited which led to resentment in some cases. Some stakeholders felt a threat to their interests and territory brought by the new processes. Where team members did not attend meetings regularly, the process of building common understanding was sometimes undermined; where new team members joined much later, they sometimes had a negative impact on the sense of safety or ‘togetherness’ in the group.

“Attendance of the group was not always 100%, and often I felt like, it’s too bad she’s not there, because last time she had some really interesting things to say and now you couldn’t follow-up on that. And then at the end of the project, adding someone new… that was not good for the sense of safety in the group.” (NL1/P2)

## Task Orientation

A higher score for the Task Orientation sub-scale is associated with a greater preparedness to discuss standards of performance, the critical appraisal of weaknesses, and a tendency to build on each other’s ideas (Table 7).

**Table 7:** Explanations for changes in the Task Orientation sub-scale.

	Where Task Orientation score increased (8 sites)	Where Task Orientation score decreased (5 sites)

**Thematic statements**	Time was devoted to jointly identifying weaknesses and potential improvements (ongoing appraisal and reflection), using feedback from the initiative evaluation Good planning and effective management was used to establish priorities, check tasks and ensure the initiative remained on track Multidisciplinary meetings were well attended and enabled professionals to openly and honestly discuss operational problems and solutions, exchange perspectives and collaborate There was good collaboration between steering group members who developed a trusting relationship, shared perspectives and jointly discussed options and decisions Operational level staff as well as higher managerial staff were present in the steering group, which assisted in the identification of improvements.	The service being improved was publicly subsidised and therefore quite defined/constricted (making it harder to question)

Factors associated with an increase in score for Task Orientation were adequate time for identifying weaknesses, strong project management, good relationships enabling honest and critical reflection, discussion and appraisal and the involvement of operational, front-line staff in decision-making roles.

“The space [time and place to meet]. I believe that having this time that we could dedicate to it has helped us, on the one hand, for organising, and the other, having those spaces to do it” (SP2/P2)

“I think that what was most facilitating were the key persons, the key person in each centre/level/sector. In particular, well the nurses that had this overall co-ordinating role. Because otherwise it is such a broad theme, isn’t it? I think… looking in from the outside that well, if it wasn’t for this person that pushes, that knows, that has the view of what we are doing and what’s next and so forth […] is not feasible.” (SP1/P38)

“When we talked to the staff one-to-one, and included them in the bigger picture, they quickly became enthusiastic.” (NL2/M2).

In the five sites where the score for this category decreased, qualitative data sometimes confirmed that there was a lack of critical appraisal of the initiatives, but there was little qualitative data that might provide explanations for this. Although sites reported that people felt free to share their views and provide constructive feedback, this was not reflected in TCI-14 scores. For one site, a high profile, nationally funded initiative made it hard to critically appraise the initiative, at a local level.

## Support for Innovation

A higher score for the Support for Innovation sub-scale is associated with searching for new ways of looking at problems, time being taken to develop ideas, and high levels of co-operation in developing and applying ideas. Qualitative data revealed a number of explanations for an increase in the Support for Innovation sub-score (Table 8). The qualitative data could not adequately explain the reasons why scores decreased or stayed the same for this sub-scale in five sites. Potential reasons for this are discussed in the next section.

**Table 8:** Explanations for changes in the Support for Innovation sub-scale.

	Where Support for Innovation increased (8 sites)

**Thematic statements**	Sufficient time to meet together and to discuss and develop ideas, and to implement the initiative Goal of the improvement initiative remained a policy focus, and therefore something that all staff mobilised around Staff worked hard within existing constraints to propose solutions of how to achieve their shared vision – they co-designed the initiative to help overcome acknowledged limitations For staff who previously did not work together, or who were being asked to adopt new methods, a gradual adjustment was made towards new ways of working ‘Champions’ within the team influenced and facilitated change by demonstrating commitment, promoting innovation with passion and persistence, bringing together groups of different professions, and developing informal networks of support Staff saw the value for the service user in the overall aims of the initiative Good support from management, who helped to resolve some of the barriers Staff were given a lot of trust and responsibility, with minimal micro-management Experiencing positive results during the implementation process helped staff to become more enthusiastic about the initiative

Key factors explaining an increase in Support for Innovation occurred within the team, such as having sufficient time to work together in a constructive way, and having a positive, solution-oriented approach; factors within organisations such as support from management, and freedom to innovate; and external factors such as alignment with policies.

Support for Innovation scores sometimes increased despite reported time constraints, staff shortages and absence of additional resources. Some initiatives required significant changes to existing services which prompted mixed feelings or attitudes from staff affected. Whilst some teams worked well together, staff felt that higher levels of commitment could have been demonstrated at institutional level, for example, by increasing capacity, by making work schedules compatible, and by supporting professionals with training.

“It is the ideal way of working. The problem is that we do not have the environment nor the usual working way allowing us to do it. It means an extra effort, extra hours…coordinating with social services, coordinating with…Of course this requires time. Work schedules need to be made compatible.” (SP1/P1)

The macro level policy and economic context made some initiatives difficult due to political uncertainty or restricted public expenditure, for example.

## Discussion

This paper explored the team climate of health and social care professional teams implementing integrated care initiatives for older people in Europe, and examined the factors contributing to team climate. Overall, team climate was high among the initiatives included in this study, and improved over time.

A shared vision and common goals have been highlighted as important for interprofessional collaboration in a number of other studies [14,15,16]. This has been described as cultural integration where norms and values are shared [17]. A key message for building a shared vision is the need to ensure integrated care initiatives are seen as valuable and feasible by the team, and developed within a reflective culture. Co-production, in which stakeholders are closely involved in developing and implementing initiatives is therefore important for success. An understanding of professional roles has been found to be important for team building and the development of structural links between health and social care services [18]. Conversely, a lack of clarity and understanding regarding professionals’ own roles and responsibilities as well as the roles of others have been described as inhibiting factors for information exchange and collaboration [18,14]. This increased knowledge of the roles of others, especially but not exclusively between health and social care organisations, seems to be strengthened as a result of the experience of working together and may not be a pre-requisite for successful teamworking. Effective leadership and good planning were identified as enablers of integrated working in this and other studies. Within organisations, support from management and the freedom to innovate were also important enablers. The need for ‘whole systems thinking’ has been highlighted in other studies and requires clear strategic vision and planning at local, regional and national level [18]. In our study, retaining a focus on improving the care of older people contributed to the development of a shared vision. This emphasis on improving quality of care has been found to be important in securing engagement and buy-in from practitioners, influencing their decision to take part in integrated care initiatives [19,20].

Along with shared vision and goals, the importance of interpersonal relationships is key. In this study, the act of designing and implementing the integrated care initiative brought professionals together, strengthened relationships, enhanced trust, and increased a sense of team identity. However, this often depends upon a small number of professionals with personal attributes of motivation, a ‘can-do’ attitude and an ability to engage others, which may make integrated care initiatives vulnerable in terms of sustainability if these key individuals leave the team.

Enablers of Participative Safety were strong inter-personal relationships, shared decision-making, high levels of commitment and motivation and the involvement of different agencies, including patient representatives. These findings are broadly consistent with the literature where teams with a more positive team climate, particularly in the domain of Participative Safety, reported more effective communication and high levels of mutual support [6,7]. A positive team climate has been described as one in which practitioners ‘work through issues’ through the creation of common bonds in an environment which is supportive, trusting, collegial and respectful and where practitioners were able to share their expertise and learn from each other [14]. This has been described as social integration where there is mutual understanding, trust, respect, and appreciation [17]. The development of a sense of friendship and community is also evident in high functioning teams [21,22]. Social and cultural integration are therefore, important elements for teamworking within complex health and social care systems.

Communication and clear decision-making processes are critical elements enhancing interprofessional collaboration and team functioning, especially when clinical decisions are validated by other team members [14,15]. Thus, joint working depends on the ability to share and exchange client information [16,17]. This often requires a cultural shift towards a willingness to share information [21]. In this study, communication and information sharing depended upon regular meetings and the use of dedicated staff to co-ordinate the flow of information. However, even where staff worked well together, the lack of shared digital systems between health and social care professionals, privacy regulations, and the lack of shared accountability hindered communication, collaboration and systematic information sharing. The development of joint organisational policies and procedures at a strategic level would help move integrated care initiatives towards a more system-wide approach to care delivery.

Organisational support from managers and co-workers has been found to be necessary in order to release time and resolve workload issues in interprofessional working [14,15]. Unwieldy policies and procedures can create further pressure on teamworking [20]. In addition, provider engagement varies depending on whether practitioners feel supported in practice [23]. In this study we found that Support for Innovation increased when staff had the ‘space’ and time to work together. However, this is increasingly difficult in climates of over-stretched and under-resourced services which are compounded by staff vacancies.

Overall, the TCI-14 data provided some limited insights into team climate before and after the implementation of integrated care initiatives at the thirteen SUSTAIN sites. However, the in-depth case study work enabled a rich picture to be developed of the many challenges involved in implementing improvements in integrated care, particularly at a time of resource constraint, and within systems and structures that were often designed for silo working. Key lessons learnt are the importance of co-production, at an operational level, in developing and implementing integrated care initiatives coupled with higher level strategic support. The value of personal relationships cannot be over-emphasised. Aligning policies and procedures within a ‘whole-system’ approach is necessary for the further development of integrated care for older people.

### Methodological and theoretical considerations

The sample completing the TCI-14 at baseline and follow-up were different due to the dynamic nature of the teams. This reflects the real-world nature of health and social care integrated teams which were fluid and dynamic across the SUSTAIN sites. The reasons for this fluidity were numerous and included organisational re-structures and staff changes as some members left the organisations and new staff were appointed. Whilst at some sites, new staff were rapidly assimilated into the existing team, for others, staff changes were seen as highly disruptive and contributed to a decrease in Vision and Participative Safety in particular. Furthermore, defining the ‘team’ was particularly challenging with the proximal working group applying across a wide, cross-organisational operational team and a closer, steering group which provided strategic direction.

There were some inconsistencies and contradictions between the TCI-14 and qualitative data that might be explained by the research design. As this was a secondary analysis of the SUSTAIN data, the TCI-14, managers and professional focus groups and interviews, meeting minutes and field notes were collected concurrently. A sequential mixed methods design [24], in which the TCI-14 scores were discussed with managers and professionals would have provided greater opportunity to relate the two data sets.

Mapping the qualitative data to the TCI-14 sub-scales was challenging in that considerable overlaps were found. A number of thematic statements seemed to explain more than one sub-scale. For example, effective leadership was related to the development of a shared Vision, Participative Safety and Support for Innovation. Similarly, having the time and space for professionals to come together to work on the initiative was an important factor in establishing and maintaining Participative Safety, Task Orientation and Support for Innovation.

The qualitative data failed to provide explanations for a decrease in Support for Innovation over time. A possible explanation could be that our data reflects a more individual rather than team perspective of support. For example, it may reflect an individual’s relationship with their line manager, individual workloads or the need for training or skills development. As a result, data derived primarily from staff focus groups may not have been sensitive enough to detect these factors. This may highlight the need to explore the barriers to innovation on a more individual level.

Given the complexity of integrated care in terms of the number of challenges and enablers affecting team climate, and given that individual factors are associated with multiple dimensions of team climate, future studies might focus on the influence of key factors on total TCI-14 scores rather than individual sub-scales. An open item on the TCI-14 where respondents are asked to identify what factors are most relevant to them in terms of team climate might identify specific, contextual factors influencing complex integrated care systems. Based on this analysis, a number of omissions are apparent in the TCI-14 particularly relating to governance procedures such as transparency of decision-making and the degree to which roles and responsibilities are clearly defined. The extent to which agreed actions are carried out may also be an important factor influencing Task Orientation.

### Limitations of the study

The small sample size at each site and the different samples completing the TCI-14 at baseline and follow-up mean the results should be interpreted with caution. It was not possible to break-down the results in terms of the different types of care as described in Table 1.

## Conclusion

The SUSTAIN project has demonstrated positive team climates in multidisciplinary, cross-organisational teams providing integrated care for older people with complex health and social care needs, in Europe. Positive team climates can be developed and maintained by providing time for exchange, understanding roles and responsibilities, building bonds of trust, and working together to pursue a common goal. However, resistances need to be identified and tackled, and agreements need to be implemented, through strong leadership, clear and accepted decision-making processes, and effective project management. Support for innovation isn’t just providing time to develop new ideas and thinking out of the box, but also making sure that changes are implemented as agreed in order to foster positive integrated care team climates and make integrated care initiatives work. The TCI-14 is a useful tool with which to measure team climate however, in order to gain a deeper understanding of the barriers and enablers of team climate, a mixed methods approach is advocated.
